# Biofilms and persistent wound infections in United States military trauma patients: a case–control analysis

**DOI:** 10.1186/1471-2334-14-190

**Published:** 2014-04-08

**Authors:** Kevin S Akers, Katrin Mende, Kristelle A Cheatle, Wendy C Zera, Xin Yu, Miriam L Beckius, Deepak Aggarwal, Ping Li, Carlos J Sanchez, Joseph C Wenke, Amy C Weintrob, David R Tribble, Clinton K Murray

**Affiliations:** 1 Extremity Trauma and Regenerative Medicine Task Area, United States Army Institute of Surgical Research, JBSA Fort Sam, Houston, TX, USA; 2 Department of Medicine, Infectious Disease Service, Brooke Army Medical Center, JBSA Fort Sam, Houston, TX, USA; 3 Infectious Disease Clinical Research Program, Uniformed Services University of the Health Sciences, Bethesda, MD, USA; 4 Department of Clinical Investigation, Brooke Army Medical Center, JBSA Fort Sam, Houston, TX, USA; 5 Walter Reed National Military Medical Center, Bethesda, MD, USA

**Keywords:** Biofilm, Risk factors, Trauma-related infections, Persistent infections, Chronic infections, Extremity wound infections

## Abstract

**Background:**

Complex traumatic injuries sustained by military personnel, particularly when involving extremities, often result in infectious complications and substantial morbidity. One factor that may further impair patient recovery is the persistence of infections. Surface-attached microbial communities, known as biofilms, may play a role in hindering the management of infections; however, clinical data associating biofilm formation with persistent or chronic infections are lacking. Therefore, we evaluated the production of bacterial biofilms as a potential risk factor for persistent infections among wounded military personnel.

**Methods:**

Bacterial isolates and clinical data from military personnel with deployment-related injuries were collected through the Trauma Infectious Disease Outcomes Study. The study population consisted of patients with diagnosed skin and soft-tissue infections. Cases (wounds with bacterial isolates of the same organism collected 14 days apart) were compared to controls (wounds with non-recurrent bacterial isolates), which were matched by organism and infectious disease syndrome. Potential risk factors for persistent infections, including biofilm formation, were examined in a univariate analysis. Data are expressed as odds ratios (OR; 95% confidence interval [CI]).

**Results:**

On a per infected wound basis, 35 cases (representing 25 patients) and 69 controls (representing 60 patients) were identified. Eight patients with multiple wounds were utilized as both cases and controls. Overall, 235 bacterial isolates were tested for biofilm formation in the case–control analysis. Biofilm formation was significantly associated with infection persistence (OR: 29.49; CI: 6.24-infinity) in a univariate analysis. Multidrug resistance (OR: 5.62; CI: 1.02-56.92), packed red blood cell transfusion requirements within the first 24 hours (OR: 1.02; CI: 1.01-1.04), operating room visits prior to and on the date of infection diagnosis (OR: 2.05; CI: 1.09-4.28), anatomical location of infected wound (OR: 5.47; CI: 1.65-23.39), and occurrence of polymicrobial infections (OR: 69.71; CI: 15.39-infinity) were also significant risk factors for persistent infections.

**Conclusions:**

We found that biofilm production by clinical strains is significantly associated with the persistence of wound infections. However, the statistical power of the analysis was limited due to the small sample size, precluding a multivariate analysis. Further data are needed to confirm biofilm formation as a risk factor for persistent wound infections.

## Background

Due to the usage of improvised explosive devices combined with the utilization of body armor protecting the abdomen and thorax during the recent conflicts in Iraq and Afghanistan (Operations Iraqi Freedom and Enduring Freedom), complex extremity wounds are prevalent among military personnel with deployment-related injuries. This injury pattern often results in infectious complications with considerable morbidity (e.g., limb amputation), extended periods of rehabilitation, and high utilization of hospital resources [1-8]. The prognosis of wounded military personnel is further impacted when infections persist or recur despite appropriate antimicrobial and surgical treatment.

Presently, few data related to infection persistence among military personnel are available [5,9]. An assessment of 664 United States (U.S.) service members with extremity injuries reported that 15% of patients developed osteomyelitis, of which 17% experienced an infection recurrence following initial treatment [5]. Among a cohort of 161 civilian patients who sustained traumatic injuries resulting in amputations, 16% had at least one rehospitalization due to an infection and 6.5% had unhealed soft-tissue at the end of the 24-month follow-up period [10]. Moreover, an analysis of 454 civilian patients with osteomyelitis, primarily resulting from soft-tissue wounds and surgical procedures, reported that 31% experienced an infection recurrence, of which 16% were determined to be relapses (original pathogen), 16% reinfections (different pathogen), and 68% could not be specified as either a relapse or reinfection [11].

In general, deployment-related complex trauma involves extensive bone and soft-tissue damage and often requires the insertion of artificial materials to maintain spatial orientation and anatomic alignment of bone fragments necessary to achieve an acceptable level of healing [7,10]. An unintended consequence of these procedures is that the medical hardware required to stabilize mangled extremities may serve as a substrate for the formation of bacterial biofilms [12-14], which has been suggested by clinicians to be a factor affecting wound healing and the persistence of infections [13]. In recent years, multiple *in vitro* models have been developed to investigate the interaction between biofilm formation and chronic wounds [15-19].

According to a National Institute of Health grant announcement issued in 2002, more than 80% of human bacterial infections are associated with biofilms [13,20]. In brief, biofilms are produced when bacteria expand horizontally and vertically on a solid surface to form a sessile, multicellular colony, which secretes a matrix of protein, polysaccharides, and extracellular DNA. The matrix allows the pathogen to circumvent the host’s immune responses and impedes the penetration of antimicrobials to the site of infection [12,21,22]. Extensive physical trauma (e.g., soft-tissue damage and vascular disruption) likely further enhances this obstruction. Moreover, bacteria in the lower stratum of the colony cease replication and become intrinsically resistant to currently approved antimicrobial agents [12,22-25]. As a result, biofilms cannot be predictably eradicated by systemic antimicrobial treatment regimens and, therefore, pose a risk for persistent wound infections, particularly when foreign bodies or medical hardware that must be retained for healing are present [12,13,23-29].

Despite the abundant literature confirming the existence of the biofilm phenomenon in *in vitro* analyses, and the increasing acceptance of this theory of disease among clinicians, an epidemiologic link has not been clearly established between the risk of wound infection persistence and the ability of pathogens to form biofilms. In the current study, our objective was to examine bacterial biofilm formation and other potential risk factors for persistent wound infections among injured military personnel in a case–control analysis.

## Methods

### Study population

Data were collected from U.S. service members with deployment-related injuries sustained between June 2009 through August 2012 and medically evacuated to Landstuhl Regional Medical Center (LRMC) for initial care before being transferred to a participating tertiary-care military treatment facility (MTF) in the U.S.: Walter Reed Army Medical Center and National Naval Medical Center in the National Capital Region (Walter Reed National Military Medical Center after September 2011), and Brooke Army Medical Center in San Antonio, TX (San Antonio Military Medical Center after September 2011). These clinical data were collected through the ongoing five-year observational cohort study of short- and long-term infectious complications following deployment-related traumatic injuries during the recent military conflicts in Iraq and Afghanistan; the U.S. Department of Defense (DoD)–Department of Veterans Affairs, Trauma Infectious Disease Outcomes Study (TIDOS). As part of the TIDOS project, a microbiological repository was established containing bacterial isolates collected from surveillance cultures at admission and cultures during the course of hospitalization [30]. This study was approved by the Infectious Disease Institutional Review Board of the Uniformed Services University of the Health Sciences (Bethesda, MD).

### Case–control identification and investigation

Information on trauma history, injury patterns, treatment, and infection characteristics for the case and control populations was prospectively collected through the DoD Trauma Registry (DoDTR, formerly the Joint Theater Trauma Registry) [31] and augmented by the TIDOS supplemental infectious disease module. Radiologic studies and clinical encounters in the medical records were retrospectively reviewed to gather data on medical hardware implantation. Bacterial isolates were obtained from the TIDOS microbiological repository. Data were collected from all levels of care: LRMC, U.S. MTFs, and post-hospitalization follow-up.

As previously described [30], infections were classified via a comprehensive review of medical records for relevant clinical findings and laboratory test results, along with the utilization of standardized definitions of the National Healthcare Safety Network (NHSN) for nosocomial infections [32]. The criterion for inclusion of wounded personnel in the study population was a diagnosis of a skin and soft-tissue infection (SSTI) and the collection of bacterial isolates from the infected wound site within one day of diagnosis (+/- 24 hours). In addition, the study population was limited to patients with SSTIs linked to one of five bacterial organisms (i.e., *Staphylococcus aureus*, *Klebsiella pneumoniae*, *Acinetobacter baumannii*, *Pseudomonas aeruginosa*, and *Escherichia coli*), reflecting clinical experience with causes of persistent wound infections in this population. Cases were identified on a per infected wound basis and required serial bacterial isolates of the same organism to be collected at least 14 days apart following initial diagnosis of an SSTI from the same infected wound. Controls (infected wounds with non-recurrent growth of bacterial isolates) were matched to cases on the basis of organism and infectious disease syndrome (i.e., SSTI). Due to the occurrence of blast injuries, patients frequently sustained multiple wounds with different microbiological growth. If more than one infected wound from a patient met the criteria for inclusion in the study population (i.e., SSTI diagnosis and isolation of specific bacterial organisms), the infected wounds were independently treated as cases and controls as appropriate. Therefore, one patient may contribute both a case and control wound to the analysis.

### Bacterial isolates and growth conditions

Bacterial isolates were recovered from frozen storage at −80°C and serially passaged twice on 5% sheep’s blood agar (Remel, Lenexa, KS) prior to experimental testing. Organisms were defined as multidrug-resistant if they exhibited resistance to at least three of the major antibiotic classes (aminoglycosides, β-lactams, carbapenems, and fluoroquinolones) or produced either extended spectrum β-lactamases or *K. pneumoniae* carbapenemases [33]. Antimicrobial susceptibility testing was performed using the BD Phoenix™ system (Becton-Dickinson, Franklin Lakes, NJ) per the manufacturer’s instructions. Susceptibility to antimicrobials was interpreted according to the criteria of the Clinical Laboratory and Standards Institute [34].

### Biofilm production

Bacterial biofilms from clinical isolates of *S. aureus*, *K. pneumoniae*, *A. baumannii*, *P. aeruginosa,* and *E. coli* were formed in 96-well plates, under static conditions for 48 hours as previously described [25]. For biofilm growth, inoculums of approximately 10^8^ CFU/mL were prepared by adjusting culture grown bacterial suspensions to an optical density (OD_600 nm_) of 0.25–0.3 and adding 100 μL of each inoculum to individual wells of a 96-well plate. After incubation for 48 hours, wells were gently washed one time with phosphate-buffered saline (PBS; pH 7.4) and stained with 100 μL of 0.1% Crystal Violet (CV) for 30 minutes at room temperature. Excess CV was removed by gentle washing with PBS, and the residual adherent CV was solubilized in 95% ethanol. Biofilm biomass was quantified by measuring absorbance of the supernatant at 570 nm. Absorbance above that level from the positive control strain, *Staphylococcus epidermidis* ATCC 12228 (ATCC, Manassas, VA), was considered positive for biofilm formation. Each strain was tested in triplicate and the mean value was recorded as the absorbance.

Among isolates of *A. baumannii*, genes for the biofilm-associated protein (*bap*) were amplified by polymerase chain reaction according to primer sequences and conditions as reported in the supplemental material (Additional file 1: Figure S1). A visual assessment of biofilm production among isolates lacking *bap*, and several representative isolates containing the *bap* gene, was performed by confocal laser scanning microscopy (Additional file 2: Figure S2).

### Pulsed-field gel electrophoresis

Clonal relationships between bacterial strains of each individual species were assessed by pulsed-field gel electrophoresis (PFGE) in accordance with the U.S. Food and Drug Administration’s method ‘Procedure for PFGE of Gram-negative rods’ (Version 1, 10/30/2007) and, as previously described [35,36], using the CHEF-DRIII system (Bio-Rad Laboratories, Hercules, CA). The endonucleases utilized for PFGE examination of *A. baumannii*, *S. aureus*, and *P. aeruginosa* isolates were ApaI, SmaI, and SpeI, respectively, while XbaI was used for *K. pneumoniae and E. coli*. Analysis of gel images was performed with BioNumerics software (Applied Maths, Austin, TX). Using previously established criteria [36,37], the PFGE patterns were interpreted and grouped into pulsed-field types (PFTs).

### Statistical analysis

Comparisons were performed using Fisher’s exact, Chi-square, and Wilcoxon Mann–Whitney tests for categorical and continuous variables, respectively. Exact conditional logistic regression was conducted to analyze the association between the potential risk factors and persistent wound infections for the univariate analysis. Independency between the SSTI case and control wounds was assumed. Data are expressed as odds ratios and 95% confidence intervals. Statistical analysis was conducted using the PROC LOGISTIC procedure from SAS® version 9.3 (SAS, Cary, NC). A value of p < 0.05 was used to define statistical significance.

## Results

### Patient demographics and characteristics

From the eligible military trauma population, 35 cases (representing 25 patients) and 69 controls (representing 60 patients) were identified. It should be noted that the 25 case and 60 control subjects included eight patients with multiple wounds were used in both groups. Patients included in the study were predominantly young men (median age of 23 years) injured via a blast mechanism (>83%) in support of Operation Enduring Freedom in Afghanistan (>88%; Table 1). The injury severity scores (i.e., a measure of overall anatomical injury severity based upon body region-specific Abbreviated Injury Severity Scale scores [38]) at admission to LRMC indicated severe trauma for both the cases and controls with median values of 24 and 21, respectively. In addition, both groups required large volume transfusions of packed red blood cells plus whole blood (PRBC) within the first 24 hours following injury (median of 34 units for cases; 18 units for controls).

**Table 1:** **Demographic characteristics and injury circumstances of deployment-injured U.S. service members (June 2009**–**August 2012)**^**1**^

**Characteristics**	**SSTI Case Patients**^**2 **^**(N = 25)**	**SSTI Control Patients**^**2 **^**(N = 60)**
*Demographics, no. (%)*		
Age, median (IQR)	22.7 (21.6, 25.5)	23.3 (21.3, 26.5)
Male	25 (100)	56 (93.3)
Enlisted	24 (96.0)	53 (88.3)
Military operation		
Operation Iraqi Freedom	0	2 (3.3)
Operation enduring Freedom	25 (100)	53 (88.3)
Unknown	0	5 (8.3)
Branch of service		
Marine	11 (44.0)	25 (41.6)
Army	12 (48.0)	26 (43.3)
Air Force/Navy	2 (8.0)	5 (8.3)
*Injury circumstance and severity*		
Blast injury, no. (%)	24 (96.0)	50 (83.3)
LRMC ISS, median (IQR)	24 (14, 29)	21 (17, 29)
Units of PRBC transfused within 1^st^ 24 hours, median (IQR)	34 (16, 83)	18 (9, 33)

IQR-interquartile range; ISS-injury severity score; LRMC-Landstuhl Regional Medical Center; PRBC-packed red blood cells plus whole blood; SSTI-skin and soft-tissue infections; U.S.-United States.

^1^Data are on a per patient basis.

^2^Eight patients with multiple infected wounds were utilized as both cases and controls.

Per the study criteria, all patients were diagnosed with skin and soft-tissue infections, of which the majority of infections (92% of case and 88% of control patients) were identified as deep soft-tissue versus superficial (Table 2). Two cases and two controls were also diagnosed with osteomyelitis. The median time following injury to the initial clinical diagnosis of the infection was 8 days for the cases and 12 days for the controls. Both groups largely completed their course of antibiotics while hospitalized (68% of cases and 78% of controls) with durations ranging from two weeks (16% and 30%, respectively) to more than 43 days (44% and 13%, respectively). Similar proportions of case (12%) and control patients (10%) experienced a surgical amputation during their hospitalization, with at least half of the procedures attributed to infections. Overall, there were three deaths among the study population (one case and two controls).

**Table 2:** **Infection characteristics and outcomes among deployment-injured U.S. service members (June 2009–August 2012)**^**1**^

**Characteristics**	**SSTI Case Patients**^**2 **^**(N = 25)**	**SSTI Control Patients**^**2 **^**(N = 60)**
Time from injury to clinical diagnosis, median days (IQR)	8.0 (5.0, 16.0)	11.5 (7.0, 21.5)
Time from injury to initial positive culture, median days (IQR)	8.0 (7.0, 17.0)	12.0 (7.0, 22.0)
*Infection type, no. (%)*^3^		
SSTI-superficial	4 (16.0)	8 (13.3)
SSTI-deep	23 (92.0)	53 (88.3)
Osteomyelitis	2 (8.0)	2 (3.3)
Foreign body	0	1 (1.7)
*Antibiotic use, no. (%)*		
1–14 days	4 (16.0)	18 (30.0)
15–23 days	3 (12.0)	16 (26.7)
24–43 days	7 (28.0)	10 (16.7)
>43 days	11 (44.0)	8 (13.3)
Completed during hospitalization^4^	17 (68.0)	47 (78.3)
Ongoing at discharge^4^	5 (20.0)	5 (8.3)
*Outcome, No. (%)*		
Limb loss during inpatient period	3 (12.0)	6 (10.0)
Limb loss due to infection	2 (8.0)	3 (5.0)
Death	1 (4.0)	2 (3.3)

IQR-interquartile range; SSTI-skin and soft-tissue infection; U.S.-United States.

^1^Data are on a per patient basis.

^2^Eight patients with multiple infected wounds were utilized as both cases and controls.

^3^Patients were diagnosed with multiple infected wounds.

^4^Data missing from one case patient.

### Wound characteristics

Extremity injuries accounted for 60% of the SSTI case wounds (37% and 23% for the lower and upper limbs, respectively), while the groin/pelvis contributed the remaining proportion. Among the SSTI control wounds, 65%, 13%, and 22% involved lower extremity, upper extremity, and non-extremity wound sites (i.e., groin/pelvis and head/chest/abdomen), respectively (Table 3). Between the two groups, there was a significant difference (p = 0.024) in the overall infection site profile. Regarding the SSTI case wounds, the occurrence of polymicrobial infections and biofilm production was significantly greater (71% and 97%, respectively; p < 0.0001) compared to the control wounds (0 and 59%, respectively). The case wounds were also predominantly characterized by multidrug-resistant organisms (MDROs); however, the proportion was not significantly different from the control wounds. Moreover, approximately 20% of both the case and control wounds required the implantation of medical hardware.

**Table 3:** **Infection characteristics among deployment-injured U.S. service members (June 2009–August 2012)**^**1**^

**Characteristics**	**SSTI Case Wounds (N = 35)**	**SSTI Control Wounds (N = 69)**	**p-value**
*Infection Location, No. (%)*			0.024
Lower extremity	13 (37.1)	45 (65.2)	
Thigh	8 (22.9)	26 (37.6)	
Gluteal muscles	0	4 (5.7)	
Knee	1 (2.8)	4 (5.7)	
Lower leg	2 (5.7)	9 (13.0)	
Ankle	0	1 (1.4)	
Foot	2 (5.7)	1 (1.4)	
Upper extremity	8 (22.9)	9 (13.0)	
Upper arm	4 (11.4)	5 (7.2)	
Forearm	4 (11.4)	4 (5.7)	
Non-extremity	14 (40.0)	15 (21.7)	
Groin/genitalia	14 (40.0)	5 (7.2)	
Head/chest/abdomen	0	10 (14.5)	
Time from injury to 1^st^ culture with growth, median days (IQR)^2^	12 (8, 38)	16 (7, 28)	0.989
OR visits prior to and on date of infection diagnosis, median (IQR)	1 (1, 1)	1 (1, 1)	0.285
Medical devices implanted in the same anatomic region as the infected wound, No. (%)	7 (20.0)	13 (18.8)	1.000
*Infection Characteristics, No. (%)*			
Biofilm production	34 (97.1)	41 (59.4)	<0.0001
Organisms susceptible to empiric antibiotics	11 (31.4)	17 (24.6)	0.154
Infections due to multidrug-resistant organisms	26 (74.3)	41 (59.4)	0.135
Polymicrobial infection	25 (71.4)	0	<0.0001

IQR-interquartile range; OR-operating room; SSTI-skin and soft-tissue infections; U.S.-United States.

^1^Data are on a per SSTI wound basis.

^2^Specific to growth of the five organisms included in the analysis: *Staphylococcus aureus*, *Klebsiella pneumoniae*, *Acinetobacter baumannii*, *Pseudomonas aeruginosa,* and *Escherichia coli.*

Overall, a total of 340 bacterial cultures were collected, of which 151 (89 from case wounds; 62 from control wounds) grew bacterial organisms and *A. baumannii* was the predominant isolate among the cases (24%) and controls (30%; Table 4). *P. aeruginosa* was also a major contributor (24% and 29% for cases and controls, respectively) followed by *E. coli* (9% and 19%, respectively), *K. pneumoniae* (8% and 1%, respectively), and *S. aureus* (3% and 6%, respectively). When the bacterial isolates were compared between the SSTI case and control wounds, there was a significant difference in the profiles (p < 0.0001). In addition, 235 bacterial isolates linked to infections and utilized in the case–control analysis (after selection through use of matching criteria) were tested for multidrug resistance (Table 5). On a per patient basis, all infections associated with *A. baumannii* and *K. pneumoniae* were due to multidrug-resistant isolates. Infections attributed to *E. coli* isolates were also largely multidrug-resistant (75% and 83% of the case and control patients, respectively). However, *S. aureus* infections only demonstrated methicillin resistance among the case patients (67%) and *P. aeruginosa* isolates were generally susceptible with both groups.

**Table 4:** **Bacterial isolates, no. (%) on a per wound basis (June 2009–August 2012)**^**1**^

	**SSTI Case wounds**	**SSTI Control wounds**	**p-value**
Bacterial organism			<0.0001
*Acinetobacter baumannii*	79 (24.0)	36 (30.3)	
*Pseudomonas aeruginosa*	78 (23.7)	35 (29.4)	
*Escherichia coli*	28 (8.5)	23 (19.3)	
*Klebsiella pneumoniae*	26 (7.9)	1 (0.8)	
*Staphylococcus aureus*	10 (3.0)	7 (5.9)	
Other	108 (32.8)	17 (14.3)	
Total Bacterial Isolates	329	119	

SSTI–skin and soft-tissue infections.

^1^Data collected from all levels of care: Landstuhl Regional Medical Center, United States military medical treatment facilities, and post-hospitalization follow-up.

**Table 5:** **Infections due to multidrug-resistant (MDR) bacterial organisms, no. (%) (June 2009–August 2012)**^**1**^

**Bacterial Organism**		**SSTI Case Patients**^**2 **^**(N = 25)**	**SSTI Control Patients**^**2 **^**(N = 60)**
*Acinetobacter baumannii*	Total Infections	9	21
	MDR	9 (100)	21 (100)
*Pseudomonas aeruginosa*	Total Infections	10	24
	MDR	0	2 (8.3)
*Escherichia coli*	Total Infections	8	12
	ESBL-producing	6 (75.0)	10 (83.3)
*Klebsiella pneumoniae*	Total Infections	1	1
	ESBL-producing	1 (100)	1 (100)
*Staphylococcus aureus*	Total Infections	3	2
	MRSA	2 (66.7)	0

ESBL-extended-spectrum β-lactamase; MRSA-methicillin-resistant *Staphylococcus aureus*; SSTI-skin and soft-tissue infections.

^1^Data are on a per patient basis and are collected from all levels of care: Landstuhl Regional Medical Center, United States military treatment facilities, and post-hospitalization follow-up.

^2^Eight patients with multiple infected wounds were utilized as both cases and controls.

### Biofilm formation

Biofilm production was assessed among the 235 isolates utilized in the case–control analysis and a significantly greater proportion of biofilms formed in association with *A. baumannii* isolates among the cases compared to the controls (p < 0.0001; Table 6). However, there were no statistical differences regarding biofilm production with either *P. aeruginosa* or *E. coli*. Data from *K. pneumoniae* and *S. aureus* were too few to be analyzed.

**Table 6:** Biofilm results, no. (%), on a per wound basis

**Bacterial Isolates**	**Biofilm Formation**	**Total**	**SSTI Case Wounds (N = 35)**	**SSTI Control Wounds (N = 69)**	**p-value**
*Acinetobacter baumannii*	Positive	19 (18.3)	12 (34.3)	7 (10.1)	<0.0001
	Negative	17 (16.4)	0	17 (24.6)	
*Pseudomonas aeruginosa*	Positive	34 (32.7)	12 (34.3)	22 (31.9)	0.543
	Negative	2 (1.9)	0	2 (2.9)	
*Escherichia coli*	Positive	11 (10.6)	6 (17.1)	5 (7.3)	0.064
	Negative	10 (9.6)	1 (2.9)	9 (13.0)	
*Klebsiella pneumoniae*	Positive	2 (1.9)	1 (2.9)	1 (1.5)	NA
	Negative	0	0	0	
*Staphylococcus aureus*	Positive	9 (8.7)	3 (8.6)	6 (8.7)	NA
	Negative	0	0	0	

NA-not applicable; SSTI-skin and soft-tissue infections.

The serial isolates of the case wounds were also examined for concordance of the PFTs and biofilm production. The majority of the case wounds had concordant PFTs with only nine cases (26%) reporting a PFT that was different from the initial isolate. In addition, bacterial isolates from 14 case wounds (40%) exhibited biofilm production that was discordant from the initially isolated sample.

Carriage of the *bap* gene was widespread in *A. baumannii*, identified in 96% of isolates (Additional file 1: Figure S1). On a per isolate basis, *bap* carriage was not associated with biofilm formation, as determined by CV uptake (p = 1.00). The three *bap*-negative isolates were recovered from infectious episodes subsequent to the initial infection with two of the isolates identified among cultures collected from the same wound site of a single patient on the same date. Each *bap*-negative isolate matched the PFGE genotype and antimicrobial susceptibility phenotype of the initial infecting isolate. Confocal microscopy images of *bap*-positive and *bap*-negative isolates suggested that *bap* may increase biofilm formation among isolates carrying this gene. Qualitatively, biofilms appeared to form among *bap*-negative isolates, but to a lesser degree than *bap*-positive isolates (Additional file 2: Figure S2).

### Univariate risk factor analysis

Site of infection (i.e., non-groin/genitalia wound versus groin/genitalia wound), biofilm production, presence of medical hardware, time from injury to first bacterial culture with growth, multidrug resistance of organisms, PRBC transfusion requirements within the first 24 hours of injury, pathogen susceptibility to empiric antibiotics, number of operating room visits prior to and on the date of clinical diagnosis, and polymicrobial infection status were examined as potential risk factors for persistent infections in an univariate analysis (Table 7). Specific bacterial organisms were not included as potential risk factors in the analysis due to their role as matching criteria for the cases and controls. Based upon the results of the univariate analysis, the following risk factors were significantly associated with persistent infections: biofilm production (p < 0.0001), multidrug resistance of organisms (p = 0.046), PRBC transfusion requirements (p < 0.001), number of operating room visitations (p = 0.022), anatomical site of infection (p = 0.003); and polymicrobial infection status (p < 0.0001). A multivariate model failed to converge due to our limited sample size.

**Table 7:** Univariate odds ratio analysis for persistent wound infection risk factors among deployment-injured U.S. service members

**Potential Clinical Risk Factor**^**1,2**^	**Odds Ratio**	**95% Confidence Interval**	**p-value**
Infection location (non-groin/pelvis versus groin/pelvis)	5.47	1.65-23.39	0.003
Time from injury to 1^st^ positive culture	1.00	0.99-1.01	0.398
Multidrug resistance of organisms^3^	5.62	1.02-56.92	0.046
Biofilm production	29.49	6.24-infinity	<0.0001
PRBC requirements within 1^st^ 24 hours^4^	1.02	1.01-1.04	<0.001
Pathogen susceptibility to empiric antibiotics	0.47	0.01-10.22	1.00
Number of OR visits prior to and on date of infection diagnosis	2.05	1.09-4.28	0.022
Polymicrobial Infection	69.71	15.39-infinity	<0.0001
Presence of medical devices in the anatomic region of the wound infection	1.05	0.35-3.17	0.925

OR-operating room; PRBC-packed red blood cells plus whole blood; U.S.-United States.

^1^Characteristics are on a per wound basis.

^2^The odds ratios for continuous predictors estimate the change in odds for a unit increase in the continuous predictor.

^3^Defined by demonstrating resistance to three or more antibiotic classes (aminoglycosides, β-lactams, carbapenems, and fluoroquinolones) or producing either extended spectrum β-lactamases or *Klebsiella pneumoniae* carbapenemases [33].

^4^Blood product transfusion data is on a per patient basis.

## Discussion

It is well-recognized that trauma-related infections often result in considerable morbidity among military personnel. Therefore, understanding potential risk factors for wound infection persistence is critical for effective infection management and to improve patient prognosis. Our analysis compared data from 35 cases (SSTI wounds with recurrent bacterial isolates at least 14 days apart) with 69 controls (SSTI wounds with non-recurrent isolates). The results of the univariate analysis suggest that infection persistence is statistically associated with biofilm formation, along with the anatomical site of the infected wound (non-groin/pelvis versus groin/pelvis), multidrug resistance of pathogens, polymicrobial infections, large volume PRBC transfusions, and the number of operating room visits leading up to the clinical diagnosis of the infection. However, pathogen susceptibility to empiric antibiotics and presence of medical hardware were not significant risk factors for persistent infections.

Despite the multitude of scientific literature available with biofilm data, there is a lack of clinical studies examining the role of biofilm formation as a risk factor for persistent wound infections. Consistent with our findings, studies assessing the relationship of biofilm formation and infection persistence in urinary tract infections demonstrated a strong correlation between these two factors [39,40]. To the best of our knowledge, the current analysis provides the first statistical data linking bacterial production of biofilms with the persistence of traumatic wound infections and corroborates earlier reports on the potential virulence of the bacterial biofilm phenotype [25]. Biofilm formation, in our analysis, was largely attributed to multidrug-resistant *A. baumannii*, which corresponds to a prior study reporting a statistical increase in the proportion of *A. baumannii* isolates that were biofilm-producing and multidrug-resistant (73%) when compared to *P. aeruginosa* (57%) [41]. In addition, a recent analysis utilized clinical isolates collected from patients including military personnel admitted to a U.S. MTF to evaluate biofilm formation. Overall, 61% of isolates produced biofilms, which were predominantly associated with MDROs, including *A. baumannii*. It was also determined that biofilm-producing isolates were more commonly collected from wound sites (e.g., bone and soft-tissue) compared to urine and blood [25].

Furthermore, the direct involvement of the *A. baumannii bap* gene in biofilm formation and maturation on medically-relevant abiotic surfaces (e.g., polypropylene, polystyrene, and titanium) has been suggested [42]. The *bap* gene carriage is widespread among *A. baumannii* isolates and may contribute to increased biofilm formation; however, in the absence of data elucidating *in vivo* gene expression, our data do not suggest carriage of this gene by infecting isolates is sufficient to constitute a risk factor for persistent infections. Moreover, confocal microscopy visualization of biofilm formation by *bap*-negative isolates suggests a redundancy of mechanisms for biofilm formation *in A. baumannii*. Nonetheless, it should be noted that this analysis was limited by the infrequency of *bap*-negative isolates.

In general, implantation of medical devices (e.g., materials for wound stabilization, catheters, and joint prosthetics) has been frequently associated with the production of biofilms and subsequent infections in both military and civilian populations [12,13,23-25,29]. Therefore, it was surprising that the presence of medical hardware was not statistically significant in the univariate model. One explanation could be the low number of wounds (e.g., open fractures) that required the implantation of medical hardware (~20%) among both the cases and controls; thus, limiting the power of the analysis. In addition, information on medical hardware was not collected prospectively, which introduces the biases and limitations related to retrospective data analysis. Furthermore, the date of medical device implantation was an estimation based upon radiology reports, so the direct association of bacterial isolates with the hardware remains uncertain. Lastly, data on medical devices in the clinical records were not specific as to exact anatomic sites (e.g., elbow and knee), but provided a more general location (e.g., limbs). As a result, it cannot be definitively known if the site of hardware implantation was the same site of infection. Nonetheless, in the absence of medical hardware, contamination of soft-tissue wounds by foreign bodies also provides a suitable substrate for biofilm growth and impacts the effectiveness of treatment. In particular, patients treated exclusively with targeted antimicrobial therapy, and not in conjunction with the removal of foreign bodies from infected sites, are likely to develop persistent infections when biofilms are involved [28].

One concern of clinicians in both military and civilian settings is that the inherent tolerance of pathogens encased in biofilms to antimicrobial agents, along with a slow growth rate, creates opportunities for the transmission of resistance markers and subsequent increased prevalence of MDROs in hospital settings. A recent analysis examined surveillance cultures collected from wounded military personnel at LRMC and U.S. MTFs and reported sustained colonization levels of multidrug-resistant gram-negative organisms over a three-year period (6.6% and 12.4%, respectively) [43]. Better understanding of biofilm-producing pathogens may provide data to improve infection control strategies and reduce the transmission of MDROs.

Moreover, infection persistence further add to the large amount of hospital resources (approximately two-thirds) necessary to manage extremity injuries among wounded military personnel during the initial inpatient period and hospital readmission. In particular, the resource cost of inpatient care and rehospitalization for military personnel with extremity injuries was estimated to be approximately $667 and $139 million, respectively, during the conflicts in Iraq and Afghanistan [7]. Interventions designed to effectively mitigate biofilm formation might significantly reduce persistence among patients with wound infections. Presently, antimicrobial methods and control agents to eradicate biofilm pathogens are generally unsuccessful [13,22].

Polymicrobial infection was identified in this study to be a significant risk factor for relapsing infection in SSTIs. In contrast to wound infections involving a single species, the synergy between microorganisms with polymicrobial infections has been shown to increase bioburden, severity of infection, increased antimicrobial resistance, and enhanced inflammatory host responses within wounds [44-47]. Although these studies did not fully address whether biofilm formation, also associated with persistence, was enhanced during polymicrobial infection, it seems probable that these interactions between organisms that influence other virulence properties would also have an effect on biofilm formation. Given the observed association between polymicrobial infection and infection persistence, future studies should evaluate interactions between organisms, such as *A. baumannii* and common co-pathogens to assess the effects that this may have on virulence properties including biofilm formation and chronicity of infection.

It should be noted there are limitations to our study that should be considered. As previously discussed, the analysis was limited by the small number of *bap*-negative isolates and wounds that required the implantation of medical hardware. In addition, the statistical power of the risk factor analysis was impacted by the overall, small sample size. Specifically, when the data were examined in a multivariate analysis, the model failed to converge due to the small number of observations within each matched strata groups when analyzed in combination with the other potential risk factors. Since cultures were obtained at the discretion of medical providers, ascertainment bias could have resulted from preferential culturing of patients with clinical infections, thus missing indolent or subacute bacterial colonization among less affected patients. However, our study by design examined patients who met the NHSN definition of clinical infection. Likewise, a misclassification bias could have occurred if control wounds were subclinically colonized with bacteria and not discovered after the initial clinical infection due to lack of culturing. Nonetheless, as elegantly demonstrated in the literature, enforcement of negative cultures among controls of a case–control study can introduce a severity of illness bias, which has a more severe effect on the odds ratios than does misclassification bias [48]. Furthermore, biofilm testing has not been standardized and the available methods do not evaluate biofilm formation in identical manners. Consequently, the results of biofilm analyses may vary depending on the methodology. Among the currently available *in vitro* biofilm models, the CV method utilized in our analysis has been widely reported in the literature and is considered to be the *de facto* gold standard [49]. Since our study examined U.S. military casualties, our findings may not be generalizable to all settings and populations; however, it is notable that other armed conflicts unfortunately have produced civilian casualties complicated by *A. baumannii* wound infections [50].

Future work should seek to confirm the role of biofilm formation by *A. baumannii* and other organisms as an independent risk factor for the persistence or relapse of wound infections. In addition, translational work is needed to bring promising compounds into the clinical setting, which target the prevention of biofilm development [51], promote their dispersal [52,53], and eliminate persister cells [54]. The potential for such approaches to eradicate biofilm-mediated infections *in vivo* has recently been demonstrated [55]. Given the breadth of human clinical infections that have been proposed to involve bacterial biofilms [13,20], substantial savings in resources and iatrogenic morbidity could be realized if such therapies allowed for current durations of antimicrobial therapy to be reduced.

## Conclusions

The results of our analysis indicate that the production of biofilms is significantly associated with the persistence of wound infections. To the best of our knowledge, these are the first data directly linking biofilm formation with persistent wound infections. These findings indicate the importance of biofilms in infection management. Further analyses with a larger study population are needed to validate our data and corroborate biofilm formation as an independent risk factor for persistent trauma-related infections. In addition, the parameters of polymicrobial infections, multidrug resistance, operating room visitations, and large volume PRBC transfusions within the first 24 hours warrant investigation as to their role in the persistence of trauma-related infections.

## Competing interests

The authors declare that they have no competing interests.

## Authors’ contributions

KSA participated in the study design, data analysis, and writing of the manuscript. KM, KAC, WCZ, XY, and MLB performed the experimental study and acquisition of data. KM, DA, PL, CJS, ACW, DRT, and CKM participated in the study design and data analysis. KM, DA, CJS, JCW, ACW, DRT, and CKM helped review the manuscript. All authors read and approved the final manuscript.

## Pre-publication history

The pre-publication history for this paper can be accessed here:

http://www.biomedcentral.com/1471-2334/14/190/prepub

## Supplementary Material

Additional file 1: Figure S1 Polymerase chain reaction (PCR) screening of the biofilm-associated protein (*bap*) gene in clinical isolates of *Acinetobacter baumannii*. Seventy-six *A. baumannii* clinical isolates were screened for the presence of the *bap* gene using primer BapF (5′ tag gga ggg tac caa tgc ag) and BapR (5′ tca tga ttt gat gct gca gcg ata a). The *bap* gene was amplified under the following conditions: 95°C for two minutes, 95°C for 30 seconds, 61°C for 30 seconds, 68°C for one minute (times 30 cycles), and 68°C for two minutes. The PCR products were separate in 1% Agarose gel. *A. baumannii* ATCC strain 17978, a *bap* positive strain, was used as the positive control. A clinical isolates of *Escherichia coli* was used as a negative control. Click here for file

Additional file 2: Figure S2 Visual analysis and comparison of biofilms formed by clinical isolates of *Acinetobacter baumannii* screened for the biofilm associated protein (*bap*) gene. Representative confocal laser microscopy images of biofilms (20×) formed by clinical strains of *A. baumannii* after 24 hours of growth in chamber slides stained with a live/dead viability stain (Molecular Probes). Clinical strains confirmed by polymerase chain reaction as *bap* positive (B-C) along with the *bap* positive control (A) formed highly dense and homogenous biofilms, whereas the *bap* negative clinical strains (D-F) formed less dense biofilms with a more heterogeneous phenotype. Click here for file
