# Supplementary figures and images for: Biofilms and persistent wound infections in United States military trauma patients: a case–control analysis
Source: BMC Infect Dis. 2014 Apr 8;14:190. doi: 10.1186/1471-2334-14-190 (PMC4234323; doi:10.1186/1471-2334-14-190)

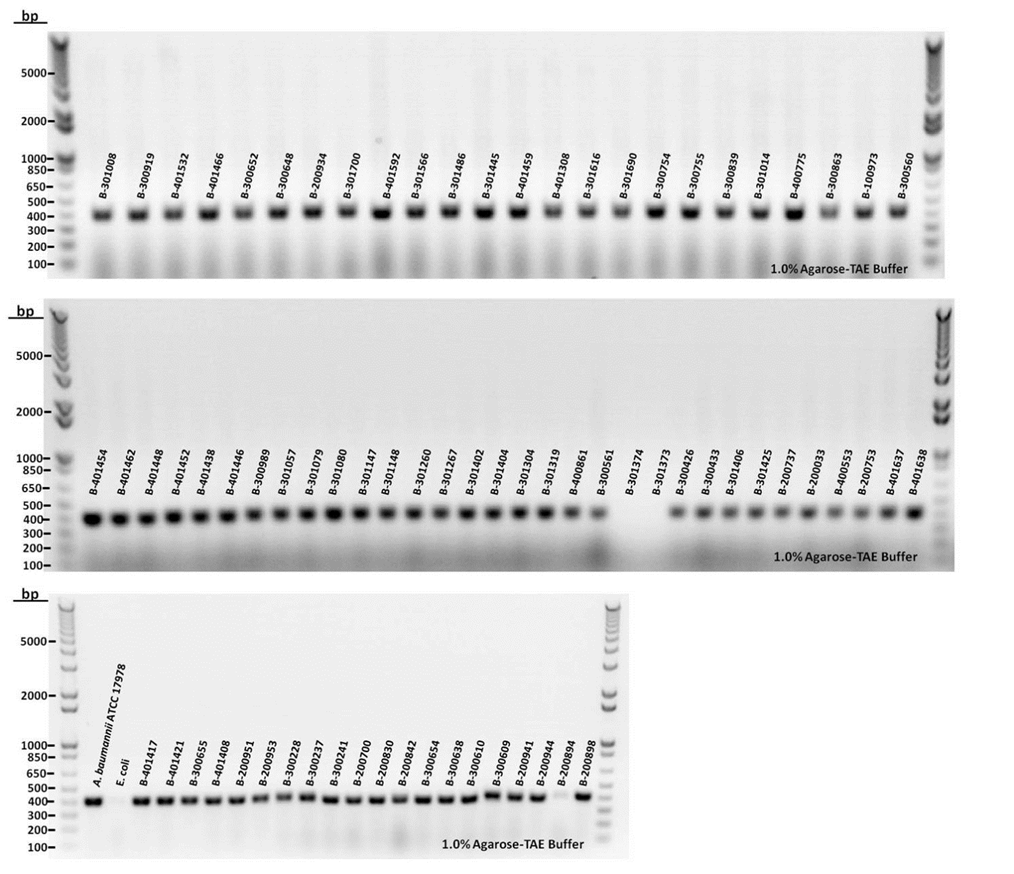

Supplement: Additional file 1: Figure S1 — Polymerase chain reaction (PCR) screening of the biofilm-associated protein (bap) gene in clinical isolates of Acinetobacter baumannii. Seventy-six A. baumannii clinical isolates were screened for the presence of the bap gene using primer BapF (5′ tag gga ggg tac caa tgc ag) and BapR (5′ tca tga ttt gat gct gca gcg ata a). The bap gene was amplified under the following conditions: 95°C for two minutes, 95°C for 30 seconds, 61°C for 30 seconds, 68°C for one minute (times 30 cycles), and 68°C for two minutes. The PCR products were separate in 1% Agarose gel. A. baumannii ATCC strain 17978, a bap positive strain, was used as the positive control. A clinical isolates of Escherichia coli was used as a negative control. [file 1471-2334-14-190-S1.tiff]

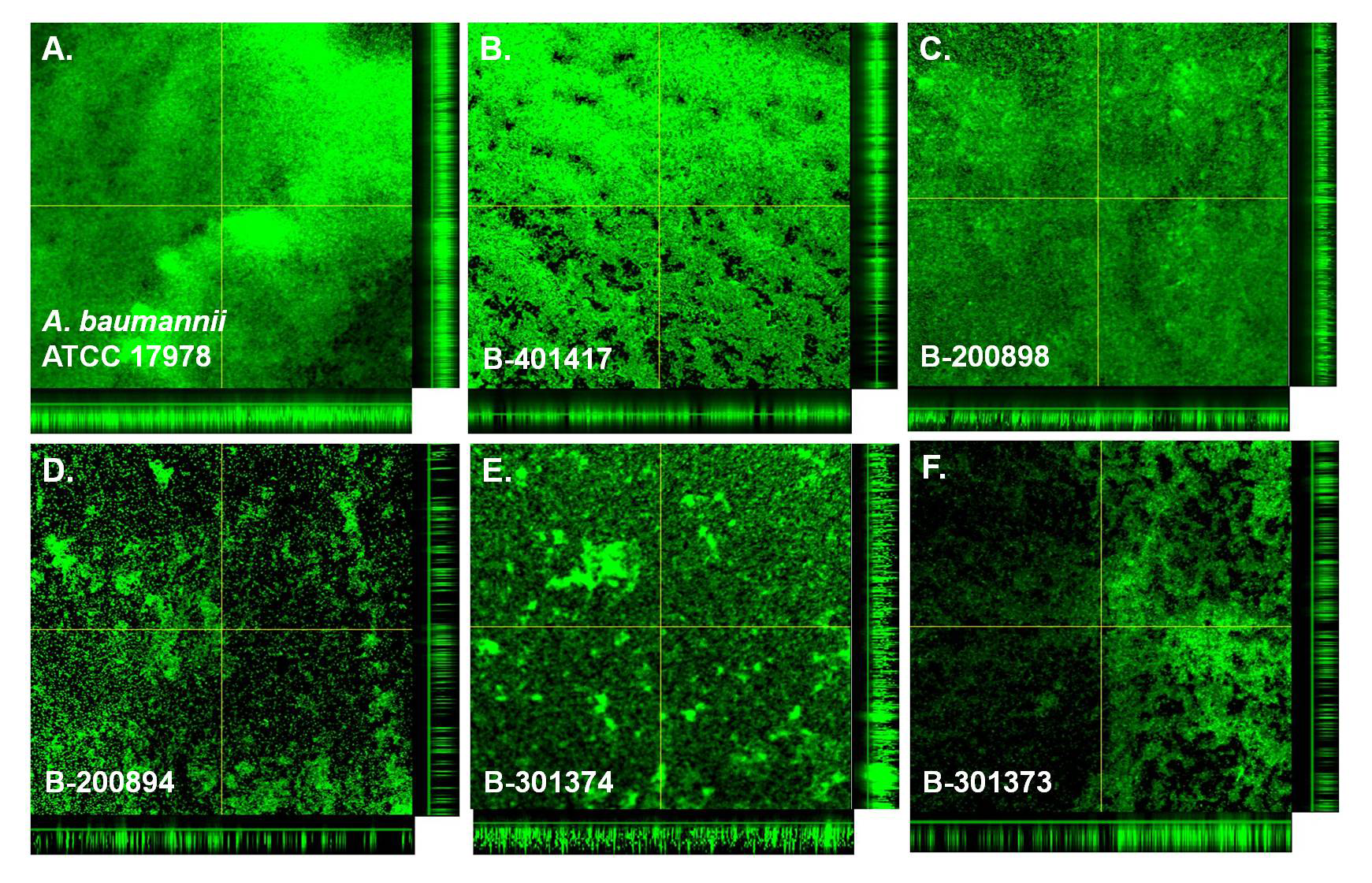

Supplement: Additional file 2: Figure S2 — Visual analysis and comparison of biofilms formed by clinical isolates of Acinetobacter baumannii screened for the biofilm associated protein (bap) gene. Representative confocal laser microscopy images of biofilms (20×) formed by clinical strains of A. baumannii after 24 hours of growth in chamber slides stained with a live/dead viability stain (Molecular Probes). Clinical strains confirmed by polymerase chain reaction as bap positive (B-C) along with the bap positive control (A) formed highly dense and homogenous biofilms, whereas the bap negative clinical strains (D-F) formed less dense biofilms with a more heterogeneous phenotype. [file 1471-2334-14-190-S2.tiff]
